# Identification and Molecular Analysis of m^6^A-circRNAs from Cashmere Goat Reveal Their Integrated Regulatory Network and Putative Functions in Secondary Hair Follicle during Anagen Stage

**DOI:** 10.3390/ani12060694

**Published:** 2022-03-10

**Authors:** Taiyu Hui, Yubo Zhu, Jincheng Shen, Man Bai, Yixing Fan, Siyu Feng, Zeying Wang, Junyin Zhao, Qi Zhang, Xingwang Liu, Tiantian Gong, Wenlin Bai

**Affiliations:** College of Animal Science and Veterinary Medicine, Shenyang Agricultural University, Shenyang 110866, China; huitaiyu0529@163.com (T.H.); 2012500032zyb@syau.edu.cn (Y.Z.); 2020220559@stu.syau.edu.cn (J.S.); 2019500012@syau.edu.cn (M.B.); fanyx@syau.edu.cn (Y.F.); 2020220555@stu.syau.edu.cn (S.F.); wangzeying2012@syau.edu.cn (Z.W.); 2019220505@stu.syau.edu.cn (J.Z.); zhangqi023@163.com (Q.Z.); xwliu987@163.com (X.L.); gongtiantian2022@163.com (T.G.)

**Keywords:** cashmere goat, M^6^A-circRNAs, secondary hair follicle, regulatory network, expression characterization

## Abstract

**Simple Summary:**

Cashmere is a natural, high-end textile material. It is derived from the secondary hair follicle (SHFs) tissue in the skin of cashmere goats. Previous studies have indicated that m^6^A modifications in circRNA molecules play important roles in a variety of cells through multiple mechanisms. However, little information is available on the expression profile and functional regulatory characteristics of m^6^A-modified circRNA (m^6^A-circRNA) in SHFs of cashmere goats. In this study, a total of 15 m^6^A-circRNAs were identified. Six of these m^6^A-circRNAs were revealed to have significantly higher expression in skin at anagen than at telogen. To gain insight into the potential regulatory mechanisms of the anagen up-regulated m^6^A-circRNAs, we constructed the regulatory networks along with related pathways in SHFs of cashmere goats. In addition, we found that the expression trends of four m^6^A-circRNAs in the SHFs during SHF cycles were highly similar to their host genes. However, the expression patterns of two m^6^A-circRNAs were inconsistent with the linear RNAs from their host genes in the SHFs of cashmere goats. These results will provide new insights to elucidate the biological functions and regulatory features of m^6^A-circRNA in SHF development and cashmere growth in goats.

**Abstract:**

*N*^6^-methyladenosine (m^6^A) is the most abundant modification in linear RNA molecules. Over the last few years, interestingly, many circRNA molecules are also found to have extensive m^6^A modification sites with temporal and spatial specific expression patterns. To date, however, little information is available concerning the expression profiling and functional regulatory characteristics of m^6^A modified circRNAs (m^6^A-circRNAs) in secondary hair follicles (SHFs) of cashmere goats. In this study, a total of fifteen m^6^A-circRNAs were identified and characterized in the skin tissue of cashmere goats. Of these, six m^6^A-circRNAs were revealed to have significantly higher expression in skin at anagen compared with those at telogen. The constructed ceRNA network indicated a complicated regulatory relationship of the six anagen up-regulated m^6^A-circRNAs through miRNA mediated pathways. Several signaling pathways implicated in the physiological processes of hair follicles were enriched based on the potential regulatory genes of the six anagen up-regulated m^6^A-circRNAs, such as TGF-beta, axon guidance, ribosome, and stem cell pluripotency regulatory pathways, suggesting the analyzed m^6^A-circRNAs might be essentially involved in SHF development and cashmere growth in cashmere goats. Further, we showed that four m^6^A-circRNAs had highly similar expression trends to their host genes in SHFs of cashmere goats including m^6^A-circRNA-ZNF638, -TULP4, -DNAJB6, and -CAT. However, the expression patterns of two m^6^A-circRNAs (m^6^A-circRNA-STAM2 and -CAAP1) were inconsistent with the linear RNAs from their host genes in the SHFs of cashmere goats. These results provide novel information for eluci-dating the biological function and regulatory characteristics of the m^6^A-circRNAs in SHF development and cashmere growth in goats.

## 1. Introduction

Cashmere, a kind of natural high-end textile material, is derived from the secondary hair follicles (SHFs) which are a dynamic mini-organ in the skin tissue of cashmere goats. The products made of cashmere, like sweaters, shawls, and scarves, are widely favored by consumers due to their unique characteristics, such as lightness, smoothness, brightness, softness, comfort and warmth retention [1]. The growth of cashmere fiber is controlled by the seasonal development of SHFs which undergo a periodic cycle (being mainly composed of three stages: anagen, catagen, and telogen) [2,3]. The growth cycle of SHFs in cashmere goats is mainly regulated by the endocrine system that changes with the photoperiod [4,5]. During the anagen stage, the SHFs are in a highly active state where many endogenous regulatory factors establish a highly coordinated regulatory network to promote the growth of cashmere fibers [6]. Therefore, it is essential to discover and characterize the novel regulatory factors implicated in cashmere growth in cashmere goats [1].

*N*^6^-methyladenosine (m^6^A) was found to be the most abundant modification in linear RNA molecules, accounting for about 80% of their methylation modifications [7,8,9]. Unlike linear RNA, circular RNA (circRNA) is formed by reverse splicing, and is a covalently closed loop without free 3′ and 5′ ends [10,11]. In recent years, it has been reported that many circRNA molecules also have extensive m^6^A modifications sites, and m^6^A modified circRNA (m^6^A-circRNA) exhibits temporal and spatial specific expression patterns during developmental processes of tissues and organs [12,13,14]. Increasing lines of evidence indicate that m^6^A modification in circRNA molecules play significant roles in a variety of cells, such as HeLa cells, liver cancer cells, and male germ cells [15,16,17,18].

Over the past few years, a considerable number of circRNAs were identified in hair follicles of cashmere goats, and many of them exhibited significantly different expression during SHF cycles [19,20,21]. Moreover, the circRNA-1926 was found to positively regulate the differentiation of SHF stem cells into hair lineages in cashmere goats through the miR-148a/b-3p/CDK19 axis [22]. More recently, the circRNA-1967 was also demonstrated to participate in the differentiation of SHF stem cells into the hair follicle lineage by sponging miR-93-3p, and as a result enhancing LEF1 expression in cashmere goats [23]. These findings suggested that circRNAs might play important regulatory roles in the development of SHFs and cashmere fiber growth in cashmere goats. Although the biological functions of the m^6^A-circRNAs in SHF development and cashmere growth need to be further clarified in cashmere goats, it is thought that the m^6^A modifications within circRNA molecules are deeply implicated in the functional roles of circRNAs through m^6^A-dependent mechanisms, such as regulating its miRNA “molecular sponge” effects [13,15,16], and its interaction with RNA binding proteins [17,18]. To date, however, little information is available concerning the expression profiling and functional regulatory characteristics of m^6^A-circRNAs in SHFs of cashmere goats. 

In this study, a total of fifteen m^6^A-circRNAs were identified and characterized for the first time in the skin tissue of cashmere goats. Of them, six m^6^A-circRNAs were revealed to have significantly higher expression in skin at anagen compared with those at telogen of the SHF cycle. Further, we generated the competitive endogenous RNA (ceRNA) regulatory network of the anagen up-regulated m^6^A-circRNAs with related pathway enrichment analysis of their potential regulatory genes through bioinformatics analysis. Also, we characterized their expression pattern along with the corresponding host genes in SHFs of cashmere goats during the SHF cycle. The results will provide new and significant information for elucidating the biological function and regulatory characteristics of the m^6^A-circRNAs in SHF development and cashmere growth in cashmere goats.

## 2. Materials and Methods

### 2.1. Sequence Source and In-Silico Analysis

All experiments were performed under approved protocol guidelines by the Animal Experimental Committee of Shenyang Agricultural University (No. 201606005). The analyzed fifteen putative m^6^A-circRNA sequences were previously obtained from full-length transcriptome sequencing data of cashmere goat anagen skin tissue (three-year-old, female, Liaoning cashmere goat breed, and body side skin) by the single-molecule real-time (SMRT) sequencing technique (unpublished data). In the full-length transcriptome sequencing analysis, The SMRTbell™ libraries were prepared following the Isoform Sequencing protocol (Iso-Seq) as described by Pacific Biosciences (PN100-092-800-03). The complementary DNA (cDNA) was synthesized using the Clontech SMARTer PCR cDNA Synthesis Kit (Clontech, Mountain View, CA, USA). Fragments >4 kb were selected using the Blue Pippin Size Selection System protocol (Sage Science, Beverly, MA, USA). The >4 kb cDNA and normal cDNAs were mixed in equimolar amounts for SMRT sequencing. Sequencing libraries were prepared using NEBNext^®^ Ultra™ RNA Library Prep Kit for Illumina^®^ (NEB, Ipswich, MA, USA) according to manufacturer’s instructions. The Illumina libraries were sequenced on the Hiseq 4000 platform (Illumina, San Diego, CA, USA). For identifying potential circRNAs from the obtained sequencing data, three popular tools were used including Find_circ [24], CIRI [25], and CIRCexplorer2 [26], and ultimately, 876 circRNAs were obtained by selecting the intersection of the analyzed results from the three identifying tools as described in our previous publication [19].

**Figure 1:**
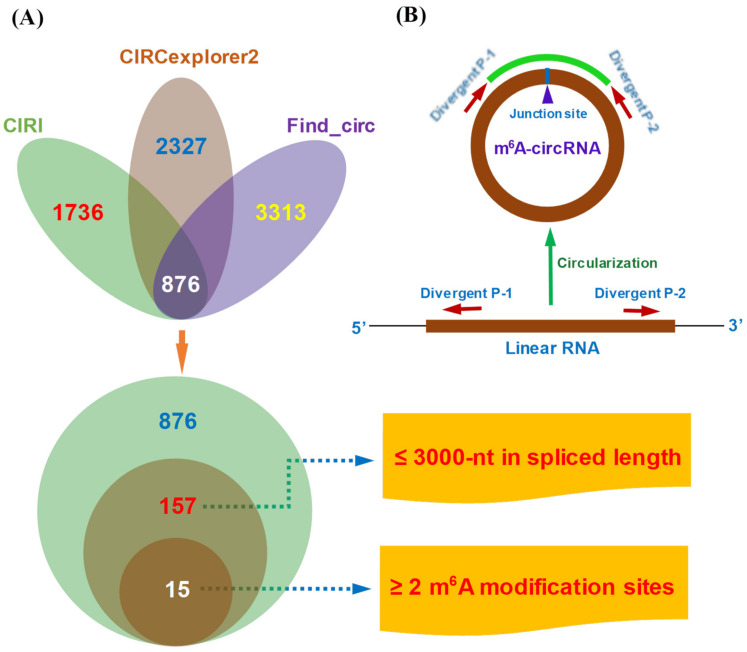
A schematic diagram of screening putative m^6^A-circRNAs with the design of divergent primer pairs. (**A**) A total of 15 putative m^6^A-circRNAs were screened out from full-length transcriptome sequencing data where the potential m^6^A sites were predicted using the SRAMP program (http://www.cuilab.cn/sramp, accessed on 11 July 2021); (**B**) The design scheme of divergent primer pairs for detecting the expression of putative m^6^A-circRNAs in skin tissue or SHFs of cashmere goat.

To our knowledge, a circRNA molecule, that is greater than 3000 nt in spliced length, often fails in its overexpression analysis for gain of function experiments in cultured cells in vitro due to the technical limitations of constructing its overexpression vector. Therefore, as shown in Figure 1A, we firstly excluded the potential circRNAs ≥ 3000-nt in spliced length from the obtained 876 circRNAs. As a result, we obtained 157 potential circRNAs less than 3000-nt in spliced length. Subsequently, we further performed a prediction for potential m^6^A sites within the 157 obtained circRNA sequences using the SRAMP program (http://www.cuilab.cn/sramp, accessed on 11 July 2021). Finally, we obtained fifteen potential m^6^A-circRNAs, each of which contained ≥2 m^6^A sites with a very high confidence in SRAMP analysis (Figure 1A). The fifteen potential m^6^A-circRNAs were selected for further analysis in the present study.

### 2.2. Skin Tissue Collection and Total RNA Isolation

Skin tissue from the side body were collected from three healthy cashmere goats (belonging to the Liaoning cashmere goat breed, that were three years old, female, and had no common traceable genetic relationships) at anagen, catagen, and telogen using sterile scalpel blades. The SHFs were further isolated from the harvested skin tissues as described in previous publications [27,28]. The total RNA was isolated from each sample using the RNAiso reagent kit (TaKaRa, Dalian, China) following the manufacturer’s instructions. The integrity of isolated total RNA was verified by agarose gel (1.5%) electrophoresis. Also, the purity and quantity of isolated total RNA were assessed through an ultraviolet spectrophotometer (Hoefer, San Francisco, CA, USA). For the ratio of OD260/OD280, all total RNA samples were verified to range from 1.8 to 2.0. Subsequently, the obtained total RNA was stored at −80 °C prior to further analysis.

### 2.3. Methylation Immunoprecipitation of m^6^A-circRNA (Me-RIP) on Skin at Anagen and Telogen

The Me-RIP of m^6^A-circRNA was performed as described by Chen and colleagues [9]. In brief, the total RNA (40 μg) was treated using RNase R (Geneseed, Guangzhou, China) and concentrated using Monarch^®^ RNA Cleanup Kit (NEB, Ipswich, MA, USA). Then, RNA was fragmented at 94 °C for 3 min with the NEBNext^®^ Magnesium RNA Fragmentation Module (NEB, Ipswich, MA, USA), and concentrated using the Monarch^®^ RNA Cleanup Kit (NEB, Ipswich, MA, USA). The 1/20 fragmented product was stored as the input control. Half fragmented RNA was incubated with 2 μg anti-m^6^A antibody (Synaptic Systems, Gottingen, Germany) or IgG (Cell Signaling Technology, Danvers, MA, USA) at 4 °C for 4 h. After pre-washing, 50 mL of the Dynabeads^®^ Protein A (Thermo Scientific, Rockford, IL, USA) was incubated with RNA-antibody complex at 4 °C for 2 h. Finally, the pulled-down RNA was isolated by the RNAiso kit (TaKaRa, Dalian, China), and the expression level of each m^6^A-circRNA was measured by the RT-qPCR technique.

### 2.4. Primer Design and RT-qPCR Analysis on Skin (Anagen and Telogen) and SHFs (Anagen, Catagen and Telogen)

In this study, a total of 24 primer pairs were used for the expression analysis of m^6^A-circRNAs along with their host genes. For the m^6^A-circRNAs, 15 divergent primer pairs were designed based on the principles shown in Figure 1B through the use of the CircPrimer program [29]. For detecting host gene expression of the six m^6^A-circRNAs (up-regulated in skin at anagen) in SHFs of cashmere goats, six convergent primer pairs were designed using the Primer Premier 5.0 program (http://www.premierbiosoft.com, accessed on 12 May 2021). In addition, the UBC, YWHAZ, and SDHA genes were utilized as combined references to normalize the RT-qPCR detection data as recommended in a previous publication [30]. Therefore, we cited their corresponding primers from the publication [30]. All primer sequences are provided in Table 1.

For detecting the expression of the m^6^A-circRNA along with their respective host genes, we conducted the RT-qPCR assay in a light Cycler 480 real-time PCR system (Roche Diagnostics, Mannheim, Germany). The reverse-transcriptions were carried out on total RNA or Me-RIP pull down RNA using the PrimeScript™ reagent with gDNA Eraser Kit (TaKaRa, Dalian, China) according to the manufacturer’s guidance. In a 25 μL final volume, RT-qPCR reactions were conducted with TB Green Premix Ex Taq II (Tli RNaseH Plus, TaKaRa, Dalian, Chnia) containing 12.5 μL TB Green Premix Ex Taq II (Tli RNaseH Plus), 1.0 μL each primer (10 μM), 2.0 μL first-strand cDNA solution, and 8.5 μL PCR-grade ddH_2_O water. The thermal cycling reactions were set as initial single cycle (95 °C for 30 s), followed by 40 cycles: 95 °C for 5 s, 52–58 °C (Table 1) for 30 s, and 72 °C for 30 s. All reactions were performed in three replicates. The m^6^A relative enrichment of each m^6^A-circRNA was normalized to the input as described in a previous publication [31], and the relative expression level of the host gene of each m^6^A-circRNA was calculated with the 2*^−ΔΔCt^* method [32].

### 2.5. Regulatory Network Analysis of Putative m^6^A-circRNAs with Related Signaling Pathway Enrichment

Accumulating evidence revealed that circRNAs could function as competitive endogenous RNAs (ceRNAs) by sharing miRNA recognition elements that relieve the expression repression of miRNA target genes [33]. Thus, we constructed the ceRNA regulatory network of m^6^A-circRNAs up-regulated in skin tissue at anagen of cashmere goats. The potential target miRNAs of m^6^A-circRNAs were predicted by the online service database: miRDB (http://www.mirdb.org, accessed on 22 May 2021). The miRDB was developed for the prediction of miRNA targets with functional annotations. According to the developers, all targets in miRDB were analyzed by the MirTarget procedure based on thousands of miRNA-target interactions from high-throughput sequencing experiments [34]. Moreover, in miRDB, common features associated with both miRNA binding and target down-regulation have been identified through a machine learning approach. Here, the custom prediction sub-procedure of miRDB was used for target miRNAs of the m^6^A-circRNAs. Due to goat data being unavailable in miRDB, human data was used to perform the predictions instead. We also predicted the potential target genes of the resultant miRNAs using the custom prediction sub-procedure of miRDB with human data. Ultimately, the ceRNA regulatory network was constructed and visualized for the analyzed m^6^A-circRNAs via the Cyotoscape (Version 2.8) procedure [35].

The miRNA mediated regulatory genes targeted by the m^6^A-circRNAs were enriched into pathways using the CluePedia built-in plugin in the Cyotoscape program with default settings (http://www.ici.upmc.fr/cluepedia/, accessed on 12 October 2021). As claimed by its developers, the CluePedia can reveal novel markers potentially associated with the pathways through calculating linear and nonlinear statistical dependencies from experimentally obtained data [36]. Ultimately, CluePedia connects the analyzed genes and integrates them into a pathway network, revealing new potential regulatory relationships among the implicated genes and signaling pathways [36].

### 2.6. Statistical Analysis

Statistical analysis was conducted with the SPSS 17.0 program (SPSS Inc., Chicago, IL, USA). The RT-qPCR data measured from different samples (*n* = 3) was presented as mean ± SD. The difference between two groups was compared with the Student’s *t*-test, and the differences among multiple groups were compared with one way analysis of variance (ANOVA) along with Bonferroni’s test. The resulting *p*-values were adjusted for multiple testing with the Benjamini–Hochberg procedure (false discovery rate, FDR adjustment,), and an adjusted *p*-value less than 0.05 was considered statistically significant. 

## 3. Results and Discussion

### 3.1. Discovery and Characterization of m^6^A-circRNAs from the Skin Tissue of Cashmere Goats with an Expression Pattern between Anagen and Telogen

**Figure 2:**
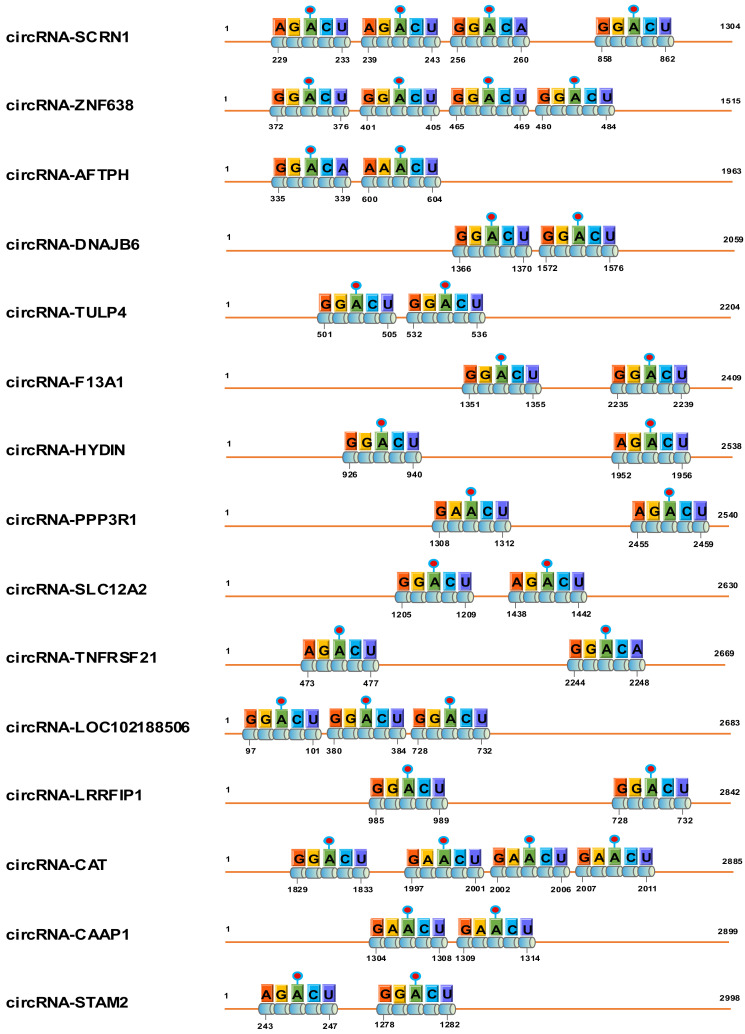
Diagram of putative m^6^A sites within 15 circRNA sequences from skin tissue of cashmere goat. The orange thin line represents circRNA sequence, and the motifs of m^6^A sites are shown within each circRNA sequence with the corresponding nucleotide positions. The putative m^6^A sites were predicted using the SRAMP program (http://www.cuilab.cn/sramp, accessed on 11 July 2021) and the only m^6^A site motifs are shown in each circRNA sequence with very high confidence in SRAMP analysis.

Over the past few years, increasing lines of evidence indicate that many circRNAs contain m^6^A modification sites with essential roles in various cells [16,17,18]. Thus, with the aim of identifying m^6^A-circRNAs from the skin tissue of cashmere goat, a total of 15 potential m^6^A-circRNAs were obtained that contained ≥ two m^6^A sites with very high confidence through SRAMP analysis (Figure 2), such as circRNA-SCRN1 containing four potential m^6^A sites, circRNA-LOC1021885063 containing three potential m^6^A sites, and circRNA-TULP4 containing two potential m^6^A sites. On the other hand, we noted that each motif of the m^6^A sites in analyzed circRNA sequences is consistent with the m^6^A site motif: RRACH (R: A/G; H: A/C/U) found in linear RNA molecules [37,38,39].

We determined the genomic information of the 15 potential m^6^A-circRNAs by mapping their sequences onto the reference genome of goat (assembly ARS1:102, https://www.ncbi.nlm.nih.gov/genome/?term=goat), accessed on 27 April 2021. As shown in Table 2, we found that several potential m^6^A-circRNAs map to the same chromosome, such as circRNA-ZNF638, circRNA-AFTPH, and circRNA-PPP3R1 on chromosome 11, as well as, circRNA-F13A1 and circRNA-TNFRSF21 on chromosome 23. The potential m^6^A-circRNAs mapped to the same chromosome are transcribed from different host genes without any overlapping region that can be defined by the mapping location information on the same chromosome. The resting eight potential m^6^A-circRNAs were mapped onto different chromosomes, such as circRNA-STAM2 on chromosome 2, circRNA-TULP4 on chromosome 9, and circRNA-CAT on chromosome 15 (Table 2).

**Figure 3:**
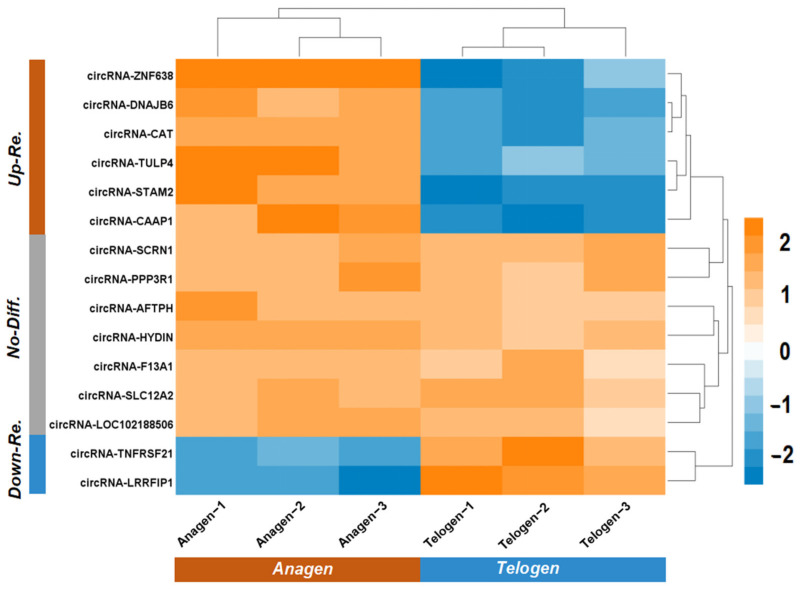
Expression analysis of 15 putative m^6^A-circRNAs in skin tissue of cashmere goats at both anagen and telogen stages presented as a hierarchical clustering heatmap. The “Up-Re” indicates the expression mean of putative m^6^A-circRNAs being significantly up-regulated in skin tissue at anagen of cashmere goats in comparison to telogen (*p* < 0.05). The “Down-Re” indicates the expression mean of putative m^6^A-circRNAs being significantly down-regulated in skin tissue at anagen of cashmere goats in comparison to telogen (*p* < 0.05). The “No-Diff” indicates no significant difference in expression mean of putative m^6^A-circRNAs in skin tissue of cashmere goats between anagen and telogen stages (*p* > 0.05). The resulting *p*-value for each comparison is presented in Table S1.

Using the Me-RIP-qPCR technique, we further analyzed the expression of the 15 putative m^6^A-circRNAs in goat skin tissue at anagen and telogen. As shown in Figure 3, the 15 analyzed m^6^A-circRNAs were verified in skin tissue of cashmere goat at both anagen and telogen. However, no significant expression difference was recorded in seven m^6^A-circRNAs between anagen and telogen, including m^6^A-circRNA-SCRN1, -F13A1, -HYDIN, -PPP3R1, -SLC12A2, -AFTPH, and -LOC102188506. The other eight m^6^A-circRNAs exhibited different expression between anagen and telogen with a significant difference (*p* < 0.05). Interestingly, there are six m^6^A-circRNAs having significantly higher expression at anagen compared with those at telogen including m^6^A-circRNA-ZNF638, -DNAJB6, -TULP4, -CAT, -CAAP1, and -STAM2 (Figure 3). In fact, the anagen phase, as a key highly active stage of cashmere goat SHFs, is rather pivotal for the morphogenesis of the cashmere fiber and its growth. During this phase, many cashmere-related molecules and regulatory factors, like inner root sheath components and various keratins, are expressed in SHFs of cashmere goat, and they coordinate closely to promote the morphogenesis and growth of cashmere fibers [19]. Therefore, we have reason to speculate that the six m^6^A-circRNAs, that exhibit significantly higher expression in anagen SHFs of cashmere goats, may be essentially implicated in the morphogenesis and growth of cashmere fibers, where they may exert their biological functions through m^6^A dependent mechanisms. This encouraged us to perform further investigations upon the six anagen up-regulated m^6^A-circRNAs, including integrated regulatory network and related pathway enrichment and expression patterns in SHFs of cashmere goat. 

### 3.2. Regulatory Network of the Up-Regulated m^6^A-circRNAs at Anagen Stage

**Figure 4:**
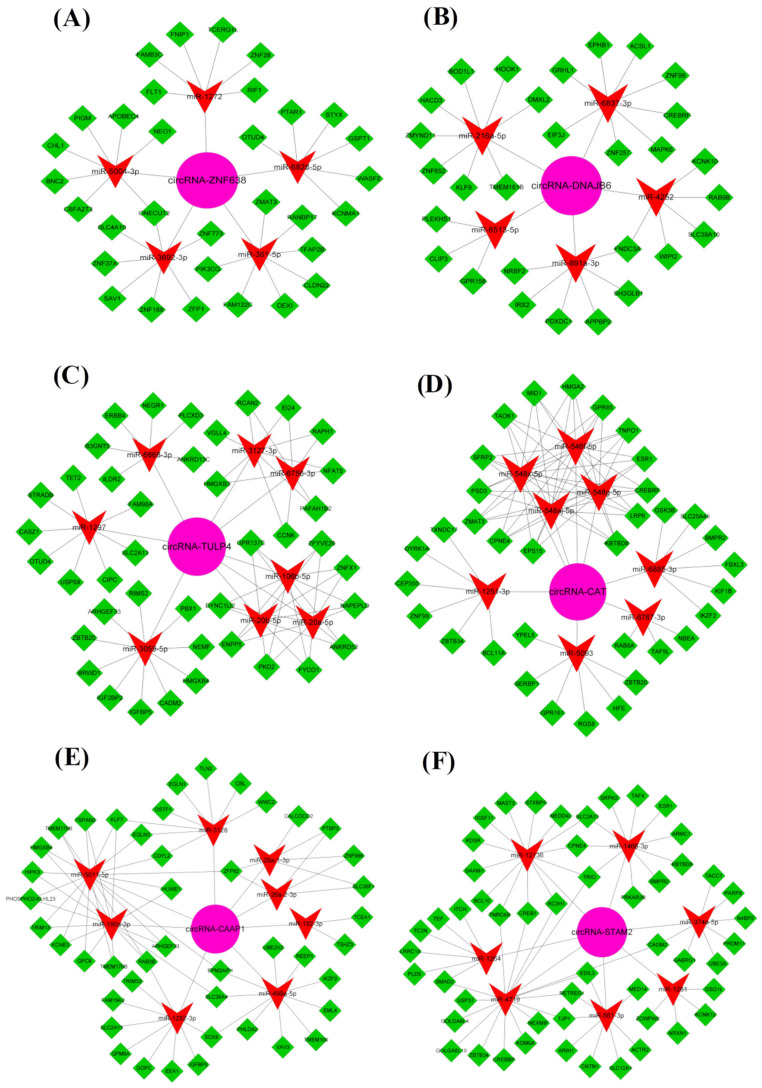
The ceRNA regulatory network of each putative m^6^A-circRNA with higher expression in anagen skin tissue of cashmere goats in comparison to telogen. The pink circle, red “V”, green diamond shapes represent m^6^A-circRNAs, miRNAs and target genes, respectively. (**A**) circRNA-ZNF638 = m^6^A-circRNA-ZNF638; (**B**) circRNA-DNAJB6 = m^6^A-circRNA-DNAJB6; (**C**) circRNA-TULP4 = m^6^A-circRNA-TULP4; (**D**) circRNA-CAT = m^6^A-circRNA-CAT; (**E**) circRNA-CAAP1 = m^6^A-circRNA-CAAP1; (**F**) circRNA-STAM2 = m^6^A-STAM2.

In recent years, studies have found that m^6^A modification in circRNA molecules can regulate the effect of circRNA as an miRNA sponge, thereby regulating the effect of miRNA on target genes. In other words, m^6^A modification in circRNA can play roles in regulating the effects of the circRNA-miRNA-mRNA axis [13,15,16]. In order to gain insight into the potential regulatory mechanism of the anagen up-regulated m^6^A-circRNAs, we constructed a competitive endogenous RNAs (ceRNAs, m^6^A-circRNA-miRNA-mRNA) network for the m^6^A-circRNAs based on in-silico analysis. As shown in Figure 4, each analyzed m^6^A-circRNA exhibited several m^6^A-circRNA-miRNA-mRNA regulatory pathways (Figure 4). As an example, the m^6^A-circRNA-ZNF638 may sponge five miRNAs (miR-1272, miR-6828-5, miR-361-5p, miR-3692-3p, and miR-5004-3p), to further regulate individually or cooperatively the expression of their target genes through a ceRNA network mechanism (Figure 4A).

**Figure 5:**
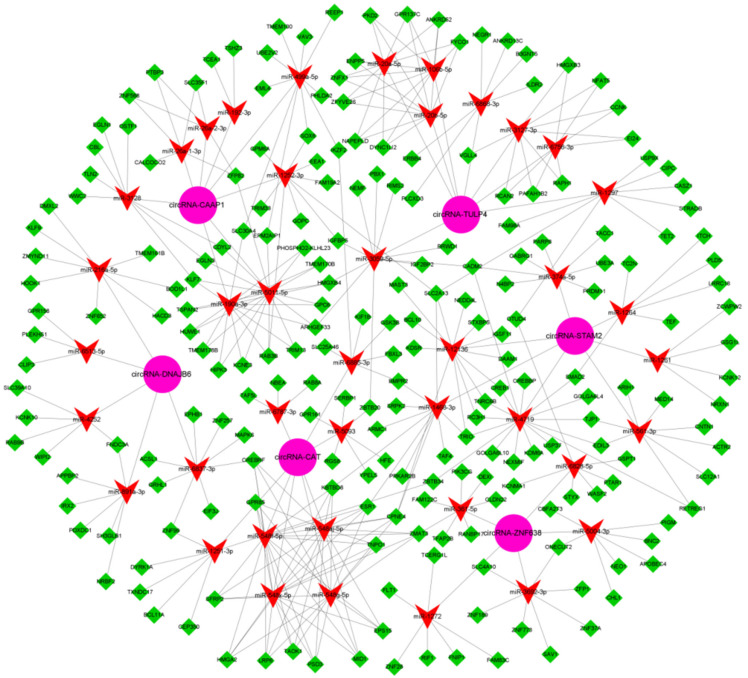
Integrated ceRNA network of the six putative m^6^A-circRNAs with higher expression in skin tissue at anagen of cashmere goats in comparison to telogen. The pink circle, red “V”, and green diamond shapes represent m^6^A-circRNAs, miRNAs and target genes, respectively. circRNA-ZNF638 = m^6^A-circRNA-ZNF638, circRNA-DNAJB6 = m^6^A-circRNA-DNAJB6, circRNA-TULP4 = m^6^A-circRNA-TULP4, circRNA-CAT = m^6^A-circRNA-CAT, circRNA-CAAP1 = m^6^A-circRNA-CAAP1, circRNA-STAM2 = m^6^A-STAM2. This integrated ceRNA network was constructed and visualized using the Cyotoscape (Version 2.8) procedure [35].

In order to gain further comprehensive insight into the integrated regulatory relationships among the different types of RNA molecules (m^6^A-circRNAs, miRNAs and mRNAs), the six fractional networks (Figure 4A–F) were merged into an integrated ceRNA regulatory network (Figure 5). Figure 5 exhibited more diverse regulatory relationships among the different types of RNA molecules. As an example, the *IGFBP5* was co-regulated by miR-3059-5p and miR-1252-3p, which were further regulated coordinately by m^6^A-circRNA-CAT and -CAAP1 (Figure 5). Interestingly, it was demonstrated that several miRNAs potentially regulated by single or multiple analyzed m^6^A-circRNAs exhibited significantly lower expression in SHFs at anagen compared with those at telogen, such as miR-20a-5p (*p* < 0.00001), miR-20b-5p (*p* = 0.00026), miR-106b-5p (*p* < 0.00001), miR-374a-5p (*p* = 0.01343), and miR-361-5p (*p* < 0.00001) [40].

On the other hand, we also found that many genes in this constructed ceRNA network were demonstrated to play important roles in the physiological processes of hair follicles. As an example, BNC2 (being potentially regulated by m^6^A-circRNA-ZNF638 through a miR-5004-3p mediated pathway, Figure 5), is mainly expressed in the matrix and the basal layer of outer root sheath at anagen, which is required for hair follicle regeneration [41]. This was similar to IGFBP5 (being potentially co-regulated by m^6^A-circRNA-TULP4 and -CAAP1 via miR-3059-5p and miR-1252-3p mediated pathways, respectively (Figure 5)). IGFBP5 was demonstrated to be a key regulator in the differentiation of hair shafts [42]. Additionally, in the constructed ceRNA network, several members of the ZNF family were revealed to be potentially regulated by the m^6^A-circRNAs via miRNA mediated pathways, such as *ZNF652* being regulated by m^6^A-circRNA-DNAJB6 via miR-216a-5p, as well as *ZNF28* being regulated by m^6^A-circRNA-ZNF638 via miR-1272. Interestingly, the ZNF family was shown to play roles in the physiological processes of cashmere goat SHFs [43]. Thus, we speculate that the m^6^A-circRNAs, act as “molecular sponges” of these miRNAs and may play essential roles in establishing an optimal expression balance of their target genes during the anagen stage of SHFs in cashmere goats. Therefore, m^6^A modifications may be required for the m^6^A-circRNAs to exert their functional significance in the morphogenesis and growth of cashmere fibers. The constructed ceRNA network provides significant information for revealing potential functional mechanisms of the m^6^A-circRNAs in anagen SHFs of cashmere goats via miRNA-mediated pathways.

### 3.3. Pathway Network of the Genes Potentially Regulated by the Anagen Up-Regulated m^6^A-circRNAs

**Figure 6:**
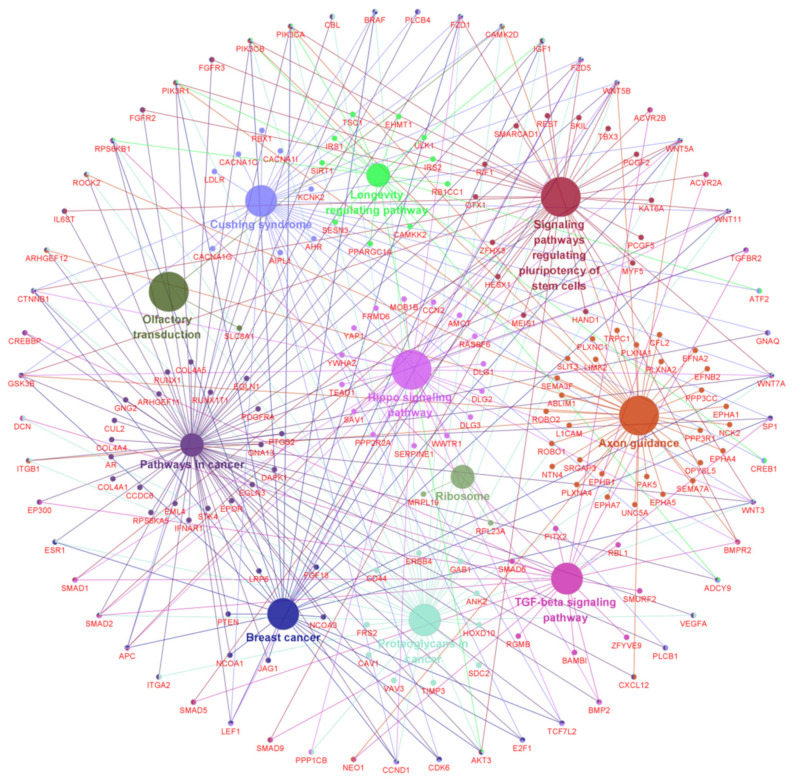
Pathway enrichment for the ceRNA regulatory genes of the six putative m^6^A-circRNAs with higher expression in anagen skin tissue of cashmere goats in comparison to telogen. The pathway enrichment was conducted using the CluePedia (a built-in plugin in Cytoscape procedure (http://www.ici.upmc.fr/cluepedia/, 12 October 2021). the enriched pathways and their corresponding genes were visualized with the same color nodes.

During anagen, the SHF physiological processes of cashmere goats is regulated by many genes that are implicated in multiple signaling pathways [2]. This means that an integrated regulatory network of related signaling pathways along with regulatory genes will contribute to revealing the functional roles and regulatory mechanisms of the anagen up-regulated m^6^A-circRNAs in cashmere goats. Based on the use of the CluePedia plugin in Cytoscape software (http://www.ici.upmc.fr/cluepedia/, accessed on 12 October 2021), we generated an integrated network of significantly enriched pathways of potential regulatory genes of the anagen up-regulated m^6^A-circRNAs (Figure 6). In Figure 6, the enriched pathways and their corresponding genes were visualized with the same color nodes. Interestingly, we noted that several enriched signaling pathways are implicated in physiological processes of hair follicles, including TGF-beta, Axon guidance, Ribosome, and Stem cell pluripotency regulatory pathways (Figure 6). For example, in previous investigations, it was recorded that the TGF-beta and mTOR signals can contribute to the activation of hair follicle stem cells by counteracting BMP-mediated suppression [44,45], whereas, the mTOR signals can be activated through ribosome S6 kinase [46]. Also, the axon guidance signals play roles in promoting the morphogenesis of hair follicles via driving the rearrangement of localized cells [47]. In addition, the stem cell pluripotency regulatory pathway is also implicated in the differentiation of hair follicle stem cells into hair follicle lineages [48]. Thus, it can be suggested that the genes potentially regulated by the m^6^A-circRNAs might be implicated in the physiological processes of hair follicles (like SHF regeneration and cashmere fiber growth), where their function might be finely regulated by the m^6^A-circRNAs through m^6^A dependent approaches.

### 3.4. Expression Patterns of m^6^A-circRNAs and Their Host Genes in SHFs of Cashmere Goats 

**Figure 7:**
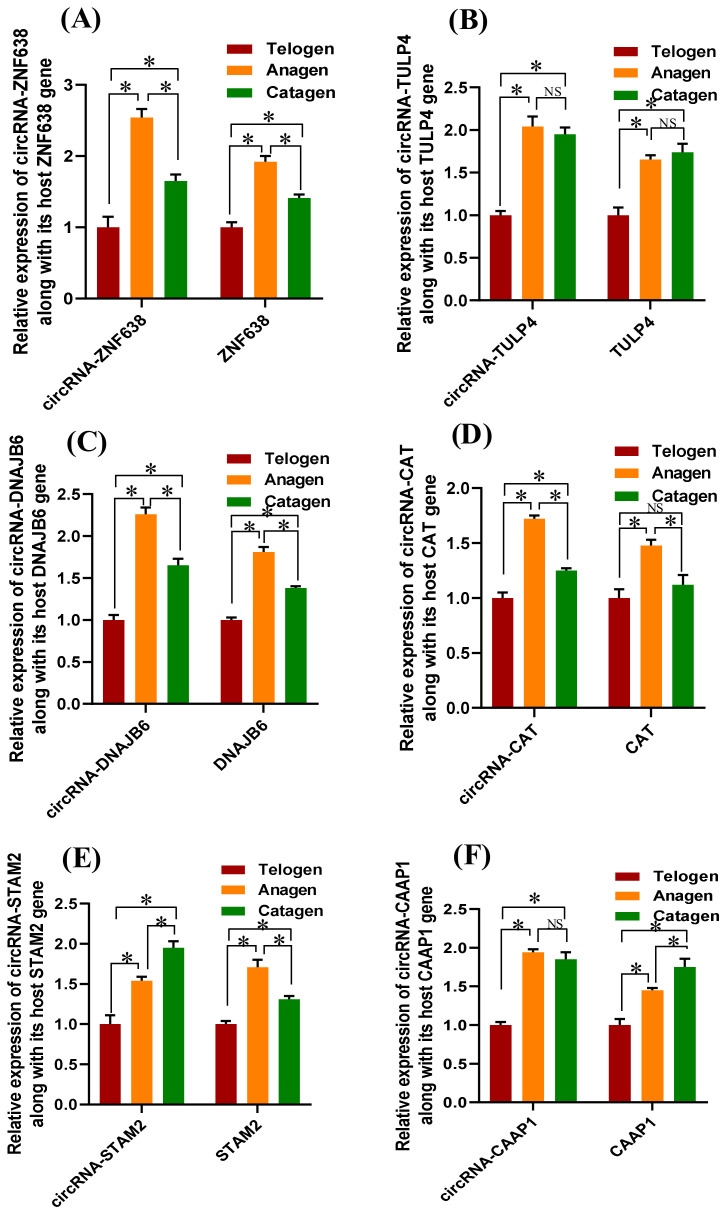
Expression analyses of the six anagen up-regulated m^6^A-circRNAs along with their host genes in SHFs of cashmere goats at telogen, anagen, and catagen stages. (**A**): circRNA-ZNF638 and its host gene ZNF638, (**B**): circRNA-TULP4 and its host gene TULP4, (**C**): circRNA-DNAJB6 and its host gene DNAJB6, (**D**): circRNA-CAT and its host gene CAT, (**E**): circRNA-STAM2 and its host gene STAM2, and (**F**): circRNA-CAAP1 and its host gene CAAP1. The expression difference was compared for each m^6^A-circRNA and corresponding host gene at telogen, anagen, and catagen, where the telogen expression data of both m^6^A-circRNA and the corresponding host gene was normalized to 1. The star symbol “*” stands for a significant difference (*p* < 0.05), and the “NS” stands for no significant difference (*p* > 0.05). The obtained *p*-value for each comparison is presented in Table S2. circRNA-ZNF638 = m^6^A-circRNA-ZNF638, circRNA-TULP4 = m^6^A-circRNA-TULP4, circRNA-DNAJB6 = m^6^A-circRNA-DNAJB6, circRNA-CAT = m^6^A-circRNA-CAT, circRNA-STAM2 = m^6^A-STAM2, and circRNA-CAAP1 = m^6^A-circRNA-CAAP1.

To gain better insight into the potential functional roles of the anagen up-regulated m6A-circRNAs in SHF development and cashmere growth in cashmere goats, we have examined their expression patterns along with the host genes in SHFs of cashmere goats during anagen, catagen and telogen stages. As observed from Figure 7, the analyzed m^6^A-circRNAs had significantly higher expression in SHFs at anagen in comparison to those at telogen, which is consistent with those recorded in skin tissue of cashmere goats (Figure 3). Additionally, the host genes of the analyzed m^6^A-circRNAs exhibited significantly higher expression in SHFs at anagen compared with those at telogen, which is highly similar to those of their corresponding m^6^A-circRNAs (Figure 7).

Our data indicated that four m^6^A-circRNAs had highly similar expression trends to their host genes including m^6^A-circRNA-ZNF638, -TULP4, -DNAJB6, and -CAT (Figure 7A–D). These observations are in line with those reported in several studies, that most circRNAs exhibited an expression pattern that is positively related with the linear RNAs from their host genes [19,49,50]. However, the expression patterns of two m^6^A-circRNAs (m^6^A-circRNA-STAM2 and -CAAP1) were inconsistent with the linear RNAs from their host genes in the SHFs of cashmere goats (Figure 7E,F). This does not seem surprising as some circRNAs were also revealed to be negatively related to the linear RNAs from their host genes in dynamic expression patterns [19,49,50].

ZNF638, the host gene of m^6^A-circRNA-ZNF638, is a member of the ZNF family. Although its biological significance in SHFs of cashmere goat is unknown, several members of this family (ZNFs) were thought to play roles in SHF physiological processes of cashmere goats including ZNF264, ZNF347, ZNF407, ZNF454, ZNF667, and ZNF704 [43]. TULP4 (the host gene of m^6^A-circRNA-TULP4) is a member of the tubby family proteins. Tubby family proteins are thought to coordinate SHH signals through their phosphoinositide-binding tubby and unique amino-terminal functional domains [51]. DNAJB6 (the host gene of m^6^A-circRNA-DNAJB6) is implicated in diverse cellular functions [52]. It was reported that DNAJB6 was involved in the activity of Wnt/β-catenin signals by controlling a regulatory loop in some manner [53]. Interestingly, SHH signaling is involved in morphogenesis and late stage differentiation of hair follicles [54]. Additionally, the Wnt/β-catenin pathway is heavily implicated in the development and induction ability of hair follicles [55,56,57]. In the present study, we showed that the expression of three m^6^A-circRNAs (m^6^A-circRNA-ZNF638, -TULP4, and -DNAJB6) and their corresponding host genes (*ZNF638*, *TULP4*, and *DNAJB6*) were significantly up-regulated at anagen in SHFs of cashmere goats (Figure 7A–C). Thus, it can be suggested that these three m^6^A-circRNAs (m^6^A-circRNA-ZNF638, -TULP4, and -DNAJB6) along with their respective host genes (*ZNF638*, *TULP4*, and *DNAJB6*) may be implicated in anagen SHF physiological processes of cashmere goats by constituting coordinated regulatory pairs. In this process, the m^6^A modifications within the circRNAs might play significant roles which may be significant for promoting SHF development and cashmere growth.

On the other hand, the anagen stage is typically characterized by the proliferation and growth of SHF cells in cashmere goats [19]. In the present study, we showed that *CAT*, *STAM2*, and *CAAP1* (being the host genes of m^6^A-CAT, -STAM2, and -CAAP1, respectively) exhibited significantly higher expression in anagen SHFs than those of telogen (Figure 7D–F). It is reported that the *CAT* gene encodes a catalase protein that is identified as a growth-promoting factor with a wide target cell spectrum including melanoma cells, myeloid leukemia cells, mastocytoma cells, as well as normal and transformed fibroblast cells [58]. *STAM2* is a phosphotyrosine protein that plays important roles in receptor signaling and trafficking of mammalian cells [59]. Although its role in hair follicle cells remains undiscovered, this protein is essentially involved in the proliferation and cell cycle of gastric cancer cells through regulating the JAK2/STAT3 signaling pathway [59]. Additionally, *CAAP1* was identified as a negative regulator of intrinsic cell apoptosis through regulating the expression and activity of the caspase protein [60], and *CAAP1* was found to promote the proliferation, migration and invasion of SMMC-7721 cells by an apoptotic inhibition of the cells [61]. Taken together with our results, it seems reasonable that *CAT*, *STAM2*, and *CAAP1* along with their corresponding m^6^A-circRNAs (m^6^A-circRNA-CAT, -STAM2, and -CAAP1) may establish a m^6^A dependent regulatory pathway in the proliferation and growth of cashmere goat SHF cells during the anagen stage.

Additionally, it should be noted that m^6^A-circRNA-STAM2 exhibited the highest significant expression at catagen (Figure 7E), and this was also the case in the expression pattern of CAAP1 among the other analyzed stages: anagen, catagen, and telogen (Figure 7F). It is well known that the catagen stage is a SHF transition stage from anagen to telogen, lasting approximately 30 days in cashmere goats, which is typically characterized by a dramatic shrinkage of the SHF lower portion along with the end of cashmere fiber growth [62]. Thus, an intriguing possibility raised is that the m^6^A-circRNA-STAM2 and CAAP1 might also be implicated in the SHF transition process from catagen to telogen in cashmere goats.

In conclusion, a total of fifteen m^6^A-circRNAs were identified and characterized from skin tissue of cashmere goats. Of them, six m^6^A-circRNAs (m^6^A-circRNA-ZNF638, -DNAJB6, -TULP4, -CAT, -CAAP1, and -STAM2) were verified to have significantly higher expression in skin tissues and SHFs at anagen compared with those at telogen. Integrated regulatory networks suggest they have potential functional roles in SHFs at anagen in cashmere goats.

## Figures and Tables

**Table 1:** Details of the primers used in this study and their real-time PCR reaction conditions.

Gene	Primer Type/Reference in GenBank or Publication	Sequence (5′–3′) ^a^	Amplicon Size (bp)	Ta ^b^ (°C)
circRNA-SCRN1	Divergent primer/this study	F: ATGCACTTTGGAAGCTGTGG, R: TGCTCAGAGTCAAGGTTGGT	246	53
circRNA-ZNF638	Divergent primer/this study	F: TTTGCAATGCCCAGGTTCAA, R: CTGACATCCCCATTCGGTCT	250	53
circRNA-AFTPH	Divergent primer/this study	F: TGCACAGTGTCTCCTTAGCA, R: ATTGTCTAGTGGTGGTGGGG	162	54
circRNA-DNAJB6	Divergent primer/this study	F: ATGAAAGAAGCCTGCACTGG, R: GGAAGTTGAAGAAGACGGCC	235	53
circRNA-TULP4	Divergent primer/this study	F: GGGACAGAAACACTCCACAG, R: CACACTCTTCAAACGGCACA	151	54
circRNA-F13A1	Divergent primer/this study	F: AACCCCGATGTCATTCAGGA, R: ACTTGCCTGTACGTGATGGA	151	55
circRNA-HYDIN	Divergent primer/this study	F: TCATCCAGCTCTCCACCAAG, R: CCCGTTTCCTCTTGCTCAAC	159	54
circRNA-PPP3R1	Divergent primer/this study	F: TCTCCATTCCCATCGGTGTC, R: CAGATAAGGATGGGGATGGA	210	54
circRNA-SLC12A2	Divergent primer/this study	F: AGGCTCAGATTGTTCTTTTGGT, R: CAAAAGCGAAGATCAGGCCA	185	53
circRNA-TNFRSF21	Divergent primer/this study	F: TGGTGATAGTGGTGTGCAGT, R: GGTCGACGTGGTGGTATTTG	234	53
circRNA-LOC102188506	Divergent primer/this study	F: GGATGAAAAGCAGAGTGGCC, R: CTGAGTTATGCCTTGGCGTC	226	54
circRNA-LRRFIP1	Divergent primer/this study	F: GAAACCACACACGGCTAAGG, R: CAGGCTGCTGAAAATGCTGA	236	54
circRNA-CAT	Divergent primer/this study	F: TCTCTCCCGGTCAAAGTGAG, R: TCGTGGCTTTGCAGTGAAAT	231	54
circRNA-CAAP1	Divergent primer/this study	F: AGGTGACAATGGTATGGACTCT, R: GCCACAGTCAGGTCTAATCC	169	53
circRNA-STAM2	Divergent primer/this study	F: TGGGTAATGTGCTGGATGGT, R: GACAACACAGCCTGCTCAAA	159	54
ZNF638	Convergent primer/XM_005686375.3	F: TTCAAGTCAAGCAGAATCCAC, R: AGACAACTCTCCCTCAACCAC	131	55
DNAJB6	Convergent primer/XM_018046635.1	F: GTTCAGTTCCTTCGGTTCGCT, R: CCTGCCGTTCACCACTTTTGT	127	57
TULP4	Convergent primer/XM_018053415.1	F: CCGACTCTTGCCTATGTTCCA, R: TCACTCCTCCCCTTTCTGCTC	165	53
CAT	Convergent primer/XM_005690077.3	F: GAGCATATTGGAAAGAGGACG, R: AAGGACGGAAACAGTAGAGCA	191	53
CAAP1	Convergent primer/XM_018051896.1	F: AACTCTACAGCCAACCTCCGC, R: ATCCCACTCCAGCAACCATCC	199	58
STAM2	Convergent primer/XM_005676127.3	F: GATGGGGATGTCTGTGGATA, R: AAAGGAGAGGCTGCTGTTGA	140	53
UBC ^c^	Convergent primer/[30]	F: GCATTGTTGGGTTCCTGTGT, R: TTTGCATTTTGACCTGTGAG	90	52
YWHAZ ^c^	Convergent primer/[30]	F: TGTAGGAGCCCGTAGGTCATCT, R: TCTCTCTGTATTCTCGAGCCATCT	102	56
SDHA ^c^	Convergent primer/[30]	F: AGCACTGGAGGAAGCACAC, R: CACAGTCGGTCTCGTTCAA	105	53

^a^ F= forward, R= reverse. ^b^ Ta = annealing temperature. ^c^ The primers were cited in a previous study (Bai et al., 2014).

**Table 2:** Genomic location information of the 15 analyzed m^6^A-circRNAs with their host genes ^a^.

circRNA Name	Spliced Length (nt)	Number of Chromosome	Sequence ID in Goat Genome	Location on Chromosome	Host Gene
circRNA-SCRN1	1304	chromosome 4	NC_030811.1	53829779 to 53831082	SCRN1
circRNA-ZNF638	1515	chromosome 11	NC_030818.1	13101422 to 13102936	ZNF638
circRNA-AFTPH	1963	chromosome 11	NC_030818.1	62684628 to 62686590	AFTPH
circRNA-DNAJB6	2059	chromosome 4	NC_030811.1	1238643 to 1240701	DNAJB6
circRNA-TULP4	2204	chromosome 9	NC_030816.1	82114863 to 82117066	TULP4
circRNA-F13A1	2409	chromosome 23	NC_030830.1	16784531 to 16786939	F13A1
circRNA-HYDIN	2538	chromosome 18	NC_030825.1	41207111 to 41209648	HYDIN
circRNA-PPP3R1	2540	chromosome 11	NC_030818.1	66320976 to 66323515	PPP3R1
circRNA-SLC12A2	2630	chromosome 7	NC_030814.1	84793912 to 84796541	SLC12A2
circRNA-TNFRSF21	2669	chromosome 23	NC_030830.1	28430416 to 28433084	TNFRSF21
circRNA-LOC102188506	2683	chromosome 10	NC_030817.1	16319392 to 16322074	LOC102188506
circRNA-LRRFIP1	2842	chromosome 3	NC_030810.1	3505854 to 3508695	LRRFIP1
circRNA-CAT	2885	chromosome 15	NC_030822.1	17940546 to 17943430	CAT
circRNA-CAAP1	2899	chromosome 8	NC_030815.1	17288789 to 17291687	CAAP1
circRNA-STAM2	2998	chromosome 2	NC_030809.1	92139671 to 92142668	STAM2

^a^ The genomic location information of the m6A-circRNAs was determined by mapping them to the goat reference genome, assembly ARS1:102, https://www.ncbi.nlm.nih.gov/genome/?term=goat, accessed on 27 April 2021.

## Data Availability

The data presented in this study are not publicly available, and the data are available on request from the corresponding author.

## Supplementary Materials

The following supporting information can be downloaded at: https://www.mdpi.com/article/10.3390/ani12060694/s1, Table S1: The resulting P value obtained from expression difference analysis of 15 m6A-circRNAs in skin tissue of cashmere goats between anange and telogen.; Table S2: The P value obtained from expression difference analysis of six anagen up-regulated m6A-circRNAs along with their host genes in SHFs of cashmere goats among anange, catagen, and telogen.
