# Supplementary material for: Identification and Molecular Analysis of m6A-circRNAs from Cashmere Goat Reveal Their Integrated Regulatory Network and Putative Functions in Secondary Hair Follicle during Anagen Stage
Source: Animals (Basel). 2022 Mar 10;12(6):694. doi: 10.3390/ani12060694 (PMC8944478; doi:10.3390/ani12060694)
Supplement: Supplementary file 1 [file animals-12-00694-s001.zip › animals-1536959-supplementary.pdf]

Table S1 The resulting *P* value obtained from expression difference analysis of 15 m^{6}A-circRNAs in skin tissue of cashmere goats between anange and telogen.

m^{6}A-circRNA name	Stages of comparison	*P*-value	The adjusted *P*-value (FDR)
circRNA-CAT	Telogen-Anagen	7.51076E-06	0.000112661*
circRNA-STAM2	Telogen-Anagen	0.000113549	0.000851619*
circRNA-DNAJB6	Telogen-Anagen	0.000123395	0.000616975*
circRNA-TULP4	Telogen-Anagen	0.00015439	0.000578962*
circRNA-CAAP1	Telogen-Anagen	0.000162429	0.000487286*
circRNA-LRRFIP1	Telogen-Anagen	0.00016981	0.000424526*
circRNA-ZNF638	Telogen-Anagen	0.000239585	0.000513397*
circRNA-TNFRSF21	Telogen-Anagen	0.000767403	0.001438881*
circRNA-HYDIN	Telogen-Anagen	0.052528163	0.087546939
circRNA-AFTPH	Telogen-Anagen	0.208914241	0.313371362
circRNA-F13A1	Telogen-Anagen	0.272032338	0.370953188
circRNA-LOC102188506	Telogen-Anagen	0.303151193	0.378938991
circRNA-PPP3R1	Telogen-Anagen	0.545204185	0.629081752
circRNA-SCRN1	Telogen-Anagen	0.702001022	0.752143952
circRNA-SLC12A2	Telogen-Anagen	0.726374136	0.726374136

* The adjusted *P*-value less than 0.05 was considered statistically significant.

Table S2 The *P* value obtained from expression difference analysis of six anagen up-regulated m^{6}A-circRNAs along with their host genes in SHFs of cashmere goats among anange, catagen, and telogen.

**circRNA-ZNF638**			**ZNF638**		
Stages of comparison	P-value	The adjusted P-value (FDR)	Stages of comparison	P-value	The adjusted P-value (FDR)
Telogen-Anagen	0.001412019	0.002118029*	Telogen-Anagen	0.000916024	0.001832048*
Anagen-Catagen	0.000409662	0.001228986*	Anagen-Catagen	0.002592168	0.007776504*
Catagen-Telogen	0.018846217	0.02261546*	Catagen-Telogen	0.019445636	0.023334763*
**circRNA-DNAJB6**			**DNAJB6**		
Stages of comparison	P-value	The adjusted P-value (FDR)	Stages of comparison	P-value	The adjusted P-value (FDR)
Telogen-Anagen	4.24556E-05	0.000127367*	Telogen-Anagen	0.000160432	0.000962592*
Anagen-Catagen	0.00083806	0.00167612*	Anagen-Catagen	0.001446811	0.008680866*
Catagen-Telogen	0.000607349	0.001822047*	Catagen-Telogen	5.2157E-05	0.000312942*
**circRNA-STAM2**			**STAM2**		
Stages of comparison	P-value	The adjusted P-value (FDR)	Stages of comparison	P-value	The adjusted P-value (FDR)
Telogen-Anagen	0.00251918	0.003023016*	Telogen-Anagen	0.000599048	0.001797144*
Anagen-Catagen	0.004839821	0.007259732*	Anagen-Catagen	0.002870837	0.005741674*
Catagen-Telogen	0.000207532	0.001245192*	Catagen-Telogen	0.010152469	0.015228704*
**circRNA-TULP4**			**TULP4**		
Stages of comparison	P-value	The adjusted P-value (FDR)	Stages of comparison	P-value	The adjusted P-value (FDR)
Telogen-Anagen	0.000405399	0.000810798*	Telogen-Anagen	0.002554705	0.003832058*
Anagen-Catagen	0.34288022	0.34288022	Anagen-Catagen	0.34952716	0.34952716
Catagen-Telogen	0.000697897	0.001395794*	Catagen-Telogen	0.000835558	0.002506674*
**circRNA-CAT**			**CAT**		

Stages of comparison	P-value	The adjusted P-value (FDR)	Stages of comparison	P-value	The adjusted P-value (FDR)
Telogen-Anagen	0.005683761	0.005683761*	Telogen-Anagen	0.009624027	0.011548832*
Anagen-Catagen	5.64577E-05	0.000338746*	Anagen-Catagen	0.007550975	0.011326463*
Catagen-Telogen	0.047633808	0.047633808*	Catagen-Telogen	0.217256598	0.217256598
**circRNA-CAAP1**			**CAAP1**		
Stages of comparison	P-value	The adjusted P-value (FDR)	Stages of comparison	P-value	The adjusted P-value (FDR)
Telogen-Anagen	6.91852E-06	4.15E-05*	Telogen-Anagen	0.013498583	0.013498583*
Anagen-Catagen	0.2196336	0.26356032	Anagen-Catagen	0.037278006	0.044733607*
Catagen-Telogen	0.002010233	0.00301535*	Catagen-Telogen	0.001148876	0.002297752*

* The adjusted *P*-value less than 0.05 was considered statistically significant.
